# Interleukin 16 contributes to gammaherpesvirus pathogenesis by inhibiting viral reactivation

**DOI:** 10.1371/journal.ppat.1008701

**Published:** 2020-07-31

**Authors:** Shuai Liu, Zhangmengxue Lei, Jie Li, Liu Wang, Ran Jia, Zhongshun Liu, Congwei Jiang, Ying Gao, Mu Liu, Linlin Kuang, Zhikang Qian, Dongming Zhou, Samuel H. Speck, Xiaozhen Liang

**Affiliations:** 1 Key Laboratory of Molecular Virology & Immunology, Institut Pasteur of Shanghai, University of Chinese Academy of Sciences, Chinese Academy of Sciences, Shanghai, China; 2 Department of Clinical Laboratory, Children's Hospital of Fudan University, Shanghai, China; 3 Department of Microbiology & Immunology, Emory Vaccine Center, Emory University School of Medicine, Atlanta, Georgia, United States of America; National Cancer Institute, UNITED STATES

## Abstract

Gammaherpesviruses have evolved various strategies to take advantage of host cellular factors or signaling pathways to establish a lifelong latent infection. Like the human gammaherpesvirus Epstein-Barr virus, murine gammaherpesvirus 68 (MHV68) establishes and maintains latency in the memory B cells during infection of laboratory mice. We have previously shown that MHV68 can immortalize fetal liver-derived B cells that induce lymphomas when injected into immunodeficient mice. Here we identify interleukin 16 (IL16) as a most abundantly expressed cytokine in MHV68-immortalized B cells and show that MHV68 infection elevates IL16 expression. IL16 is not important for MHV68 lytic infection but plays a critical role in MHV68 reactivation from latency. IL16 deficiency increases MHV68 lytic gene expression in MHV68-immortalized B cells and enhances reactivation from splenic latency. Correlatively, IL16 deficiency increases the frequency of MHV68-infected plasma cells that can be attributed to enhanced MHV68 reactivation. Furthermore, similar to TPA-mediated lytic replication of Kaposi's sarcoma-associated herpesvirus, IL16 deficiency markedly induces Tyr705 STAT3 de-phosphorylation and elevates p21 expression, which can be counteracted by the tyrosine phosphatase inhibitor orthovanadate. Importantly, orthovanadate strongly blocks MHV68 lytic gene expression mediated by IL16 deficiency. These data demonstrate that virus-induced IL16 does not directly participate in MHV68 lytic replication, but rather inhibits virus reactivation to facilitate latent infection, in part through the STAT3-p21 axis.

## Author summary

Gammaherpesviruses establish life-long infection in B cells through the regulation of virus-host interaction. Following initial lytic infection, viruses infect B cells and take advantage of host cellular factors and signaling pathways to manipulate B cell responses, ultimately establish latency in B cells, which can be reactivated to induce lytic replication in some circumstances. Here we use a mouse model of gammaherpesvirus infection and show that IL16, one unique cytokine regulating CD4^+^ T cell function, is highly abundant in gammaherpesvirus-associated lymphoma cells and can be induced by gammaherpesvirus infection. In the absence of IL16, virus reactivation from B cells is markedly enhanced and the frequency of virus-infected plasma cells that account for virus reactivation is also significantly increased. These results illustrate how gammaherpesvirus takes advantage of host cellular factor to regulate its life-long latent infection.

## Introduction

Interleukin 16 (IL16), initially identified as lymphocyte chemoattractant factor, is a novel interleukin with no significant homology to other interleukins and cytokines [1]. It is constitutively expressed in a variety of cells, such as T cells, B cells, mast cells, eosinophils, and epithelial cells [1–6]. Human IL16 is initially translated into a 631 amino acid precursor protein that can be cleaved to generate an N-terminal pro-IL16 and a 121-residue C-terminal peptide, the cleaved C-terminal peptide is subsequently released into supernatant to become aggregate and bioactive form of mature IL16 [7]. The N-terminal pro-IL16 has been shown to induce cell cycle arrest and suppress T cell growth by stabilizing the cyclin-dependent kinase inhibitor p27 [8, 9]. The IL16 gene is highly conserved within all species. Human IL16 has over 90% homology to non-human primates, 75% homology to the N terminus of mouse IL16 and 82% homology to the C terminus of mouse IL16 [10, 11].

Because the early study has revealed that IL16 can bind to CD4, the main focus of IL16 function has been investigated in CD4^+^ lymphocytes. It has been demonstrated that IL16 can induce expression of IL2 receptor alpha and beta, and synergize with IL2 to augment CD4^+^ T cell activation and proliferation [1, 12, 13]. However, the pretreatment of IL16 inhibits CD3/T cell receptor-mediated lymphocyte activation and proliferation [14]. As a chemoattractant factor, IL16 has been shown to induce migration in CD4^+^ lymphocytes, monocytes, and eosinophils [1], but *in vivo* mouse study demonstrates that CD4 is not required for IL16 function in chemotaxis and production of proinflammatory cytokine [15], suggesting the existence of alternative IL16 receptor other than CD4. The difference observed between *in vitro* and *in vivo* studies implies the complexity of IL16 function in CD4^+^ T cells.

Given the association of IL16 with CD4 that is a primary cellular receptor for HIV-1 entry, the role of IL16 in HIV-1 infection has been extensively studied. IL16 is shown to suppress the replication of HIV-1 in primary CD4^+^ T cells [16], but not the replication of HIV-1 in naturally infected peripheral blood mononuclear cells [17]. IL16 can repress HIV-1 promoter activity and viral transcription, providing a therapeutic value in HIV-1 infection [18–20]. Other than HIV-1, IL16 expression has also been linked to other infectious diseases, such as human respiratory syncytial virus, severe acute respiratory syndrome-coronavirus, and *mycobacterium tuberculosis* infection [21–23]. Additionally, IL16 promotes Tropheryma whipplei replication and is associated with Whipple's disease [24].

Human gammaherpesviruses including Epstein-Barr virus (EBV) and Kaposi sarcoma-associated herpesvirus (KSHV) are tightly associated with lymphoproliferative diseases and other cancers. Given the species-restrictive host tropism of human gammaherpesviruses, murine gammaherpesvirus 68 (MHV68) offers a unique model to define gammaherpesviral pathogenesis [25]. MHV68 infection of laboratory mice by intranasal inoculation leads to acute replication in the lung that is cleared by 9–12 days post-infection [26], followed by the establishment of latency in the spleen that predominantly involves B cells [27, 28]. Like KSHV and EBV, MHV68 can be reactivated from latently infected B cells by various stimuli, such as phorbol esters, sodium butyrate, and anti-immunoglobulin (anti-Ig).

MHV68 immortalization of fetal liver-derived B cells recapitulates the characteristic of human gammaherpesviruses that mainly induce lymphomas in immunodeficient hosts, not in immunocompetent hosts [29]. To further define MHV68-associated lymphomagenesis, in the current study, we performed cytokine array analyses and found that IL16 was highly abundant in MHV68-immortalized B cells. We further confirmed that MHV68 infection of murine primary embryonic fibroblasts (MEFs) induced IL16 expression *in vitro*, MHV68 infection of mice elevated serum IL16 level *in vivo*. Both *in vitro* and *in vivo* studies showed that IL16 had no significant role in MHV68 lytic replication, but was important for the latency maintenance by inhibiting MHV68 reactivation. IL16 regulated the de-phosphorylation of Tyr 705 STAT3 and expression of p21, consequently contributing to IL16 inhibition of MHV68 reactivation. Our results illustrate an exquisite virus-host interaction, MHV68-induced cellular IL16 helps to maintain MHV68 latency and ultimately might facilitate MHV68-associated lymphomagenesis.

## Results

### MHV68 infection induces IL16 expression

**Fig 1:**
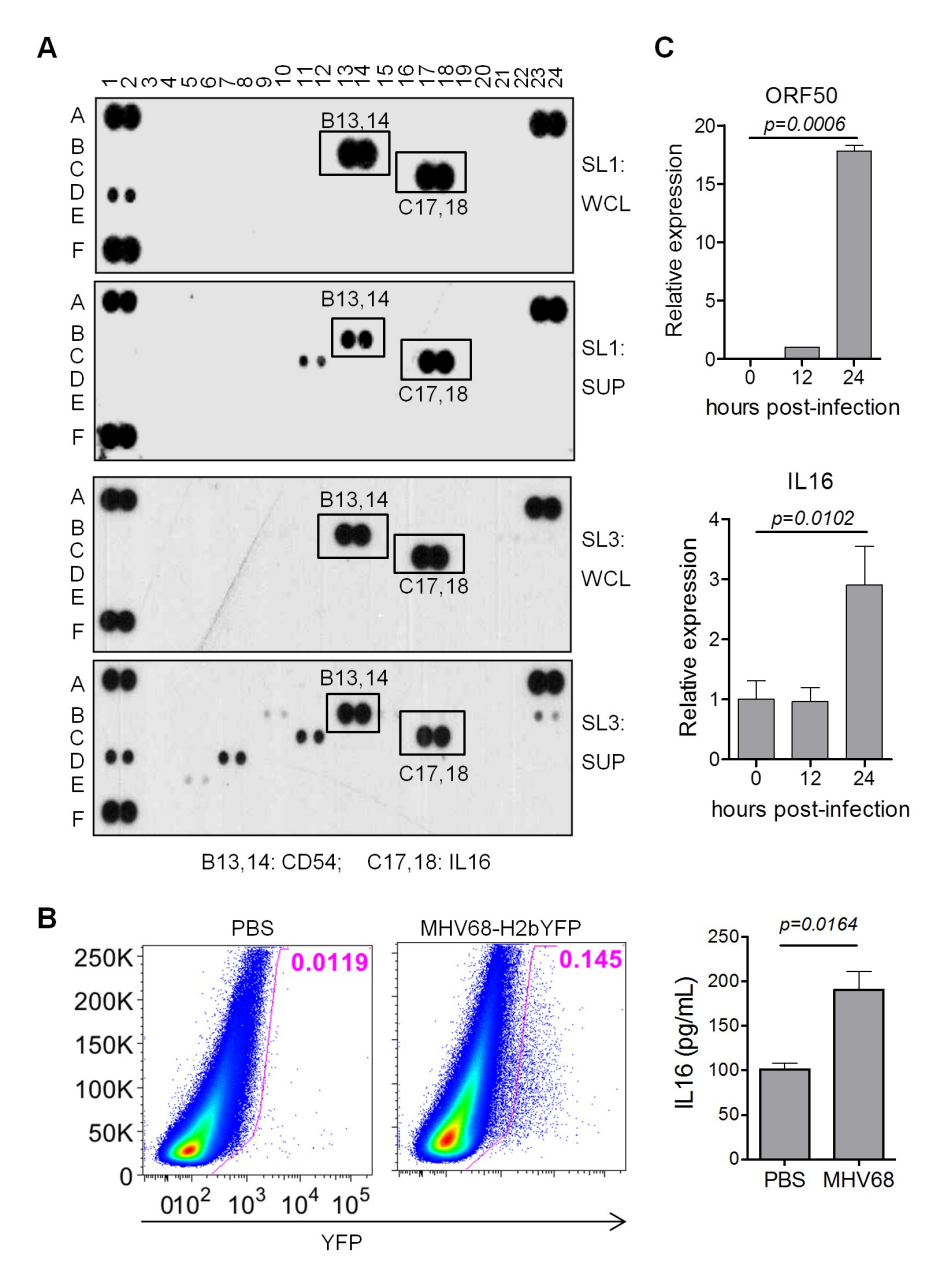
MHV68 infection increases IL16 expression. (A) 1×10^6^ SL-1 and SL-3 cells were cultured for 24 hr, whole-cell lysate (WCL) and supernatant (SUP) were prepared and subjected to cytokine array analyses. Each cytokine has one pair of duplicate spots. A1-2, A23-24, and F1-2 are experimental positive control, and F23-24 is an experimental negative control. (B) C57BL/6 mice were intranasally inoculated with 5×10^4^ PFU of MHV68-H2bYFP or mock inoculated with PBS. At day 16 post-infection, splenocytes were isolated and subjected to flow cytometry, the flow plot represented the strategy gating YFP+ MHV68 infected cells (left panel); serum was prepared from 10 virus-infected mice or mock-infected mice, followed by IL16 ELISA assay (right panel). Histograms represented mean ±SD of 10 individual mice (two experiments, n = 5 for each experiment). *p* value was determined by two-tailed unpaired t-test. (C) WT MEFs were infected with MHV68 at an MOI of 1, total RNA was isolated from infected cells harvested at the indicated time points and subjected to qRT-PCR analyses with specific primers corresponding to IL16 and MHV68 ORF50 gene. The relative RNA amount was normalized to GAPDH in each sample. Histograms represented the mean of three independent biological replicates ±SD, *p* value was determined by two-tailed unpaired t-test, *p*≤ 0.05 represents significance.

We have previously immortalized fetal liver-derived B cells with MHV68 and established an MHV68-associated lymphoma model [29]. To further define the molecular mechanism of MHV68-associated lymphomas, cytokine array analyses were performed with MHV68-immortalized SL-1 and SL-3 cells. Surprisingly, IL16, an immunomodulatory cytokine, was most abundantly present in both supernatant and cell-associated lysates of SL-1 and SL-3 cell culture (Fig 1A). Given the previous report that EBV EBNA2 could induce the expression of IL16 [30], and human B cell lines constitutively express and secrete IL16 [3], detection of a high level of IL16 expression in MHV68-immortalized cells implicates that MHV68 infection of B cells might induce IL16 expression. To confirm this, MHV68-H2bYFP reporter virus, which can efficiently track MHV68 latently infected cells [31], was used to infect C57BL/6 mice intranasally. MHV68-infected cells were gated as YFP-positive cells by flow cytometry analyses and MHV68 infection resulted in elevated serum IL16 level at day 16 post-infection (Fig 1B). Furthermore, MHV68 infection of MEFs at an MOI of 1 increased IL16 expression at 24 hr post-infection, MHV68 infection was evidenced by the detection of ORF50 expression (Fig 1C). Taken together, these data illustrate that MHV68 infection can elevate IL16 expression and secretion.

### IL16 does not affect MHV68 lytic infection

**Fig 2:**
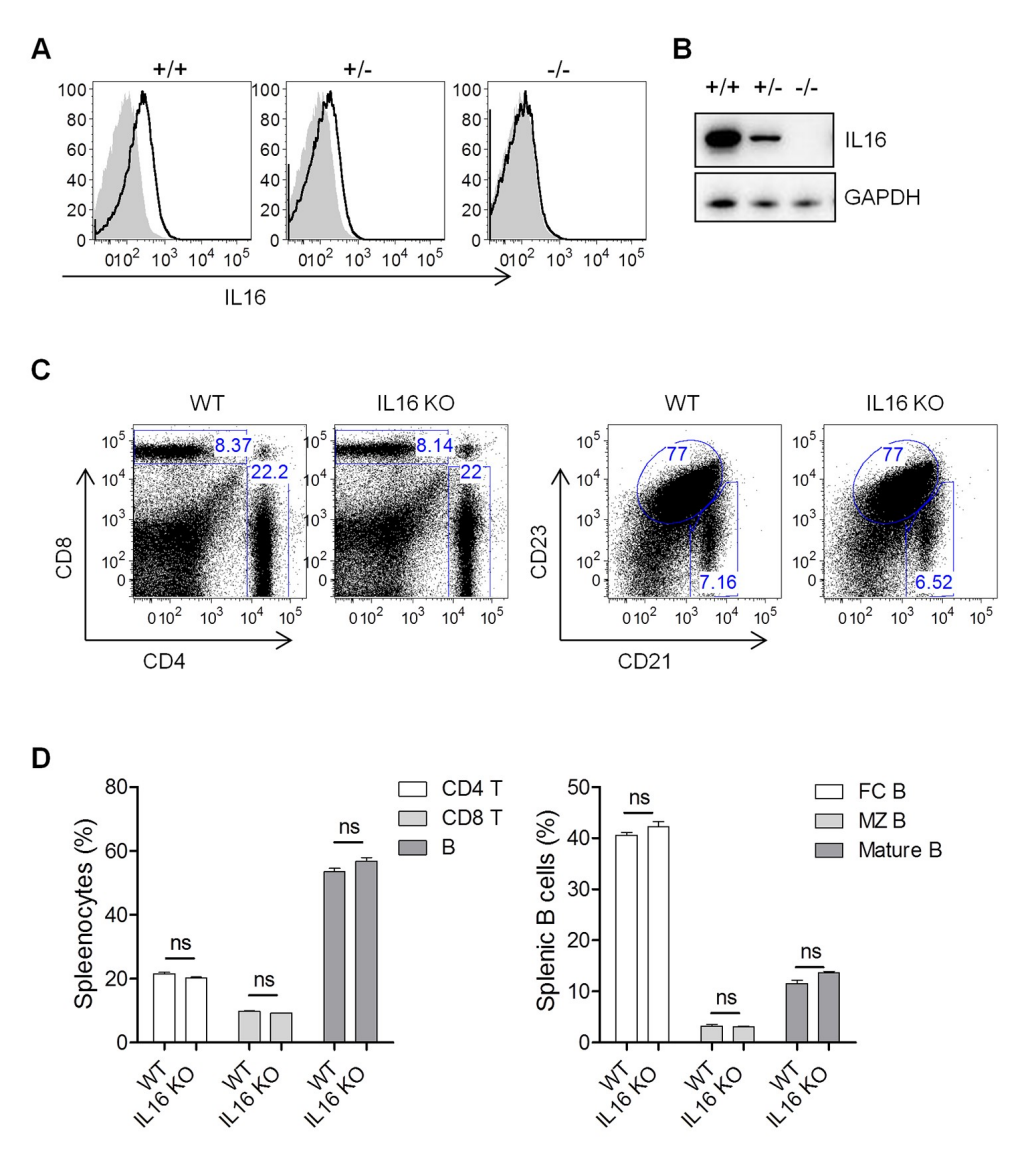
IL16 KO mice show normal B cell development. (A) Intracellular staining of IL16 in splenocytes isolated from IL16+/+, IL16+/-, and IL16-/- mice. (B) Immunoblot detection of IL16 expression in splenocytes isolated from IL16+/+, IL16+/-, and IL16-/- mice. (C) Representative flow plots showed flow cytometric analyses of splenocytes from IL16+/+ (WT) and IL16-/- (KO) mice. (D) The statistic analyses of CD4+ T, CD8+ T, and B cells (Left panel); follicular (FC) B, marginal zone (MZ) B, and mature B cells (Right panel) in splenocytes from WT and IL16 KO mice. Histograms represented mean ±SD of 8 individual mice (two experiments, n = 4 for each experiment). ns = not significant.

We next addressed whether IL16 expression has any important roles in MHV68 infection. IL16 knockout (KO) mice were generated with the CRISPR-Cas9 genome-editing system by targeting IL16 exons 2 and 3. The genotyping and sequencing analyses confirmed the deletion of 2247 bp across IL16 exons 2 and 3 regions. The intracellular staining and immunoblot analyses revealed the efficient knockout of IL16 gene expression (Fig 2A and 2B). Both B and T cells exhibited normal development and differentiation in the spleens of IL16 KO mice, showing similar frequency and number of CD4 T, CD8 T, B, mature B, follicular B, and marginal zone B cells to wild-type (WT) mice (Fig 2C and 2D).

**Fig 3:**
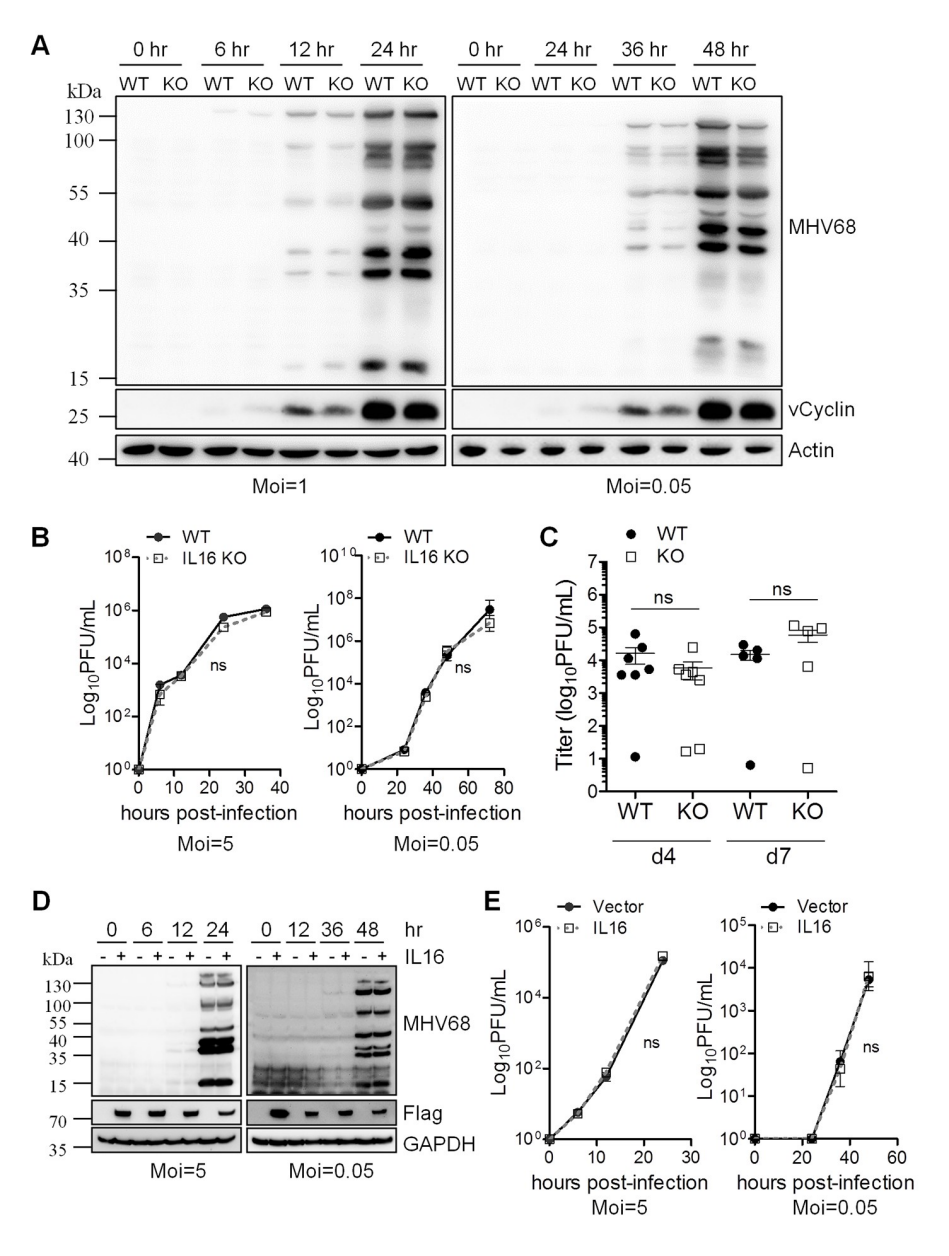
IL16 does not affect MHV68 lytic infection. (A) WT and IL16 KO MEFs were infected with MHV68 at an MOI of 1 or 0.05. The infected cells were harvested at the indicated time points and immunoblot analyses were performed with specific antibodies as indicated. Actin was used as a loading control. (B) WT and IL16 KO MEFs were infected with MHV68 at an MOI of 5 or 0.05. The supernatant was harvested at the indicated times and viral titers were determined by TCID50 assay. Results are means from triplicate samples. Error bars represented standard deviations. ns = not significant. (C) WT and IL16 KO mice were intranasally infected with 5×10^4^ PFU of MHV68. Lungs of infected mice were collected at day 4 and 7 post-infection. Virus titers were determined by TCID50 assay. Data represented one of two independent experiments with 5 or 7 mice per group. ns = not significant. Each symbol represented an individual mouse. The horizon line indicated geometric mean titer. (D) Vector or IL16-expressing plasmids with Flag tag were transfected into BHK21 cells for 24 hr, followed by MHV68 infection at an MOI of 5 or 0.05. The infected cells were harvested at the indicated time points and immunoblot analyses were performed with specific antibodies as indicated.–and + represents the cells transfected with vector and IL16-expressing plasmids with Flag tag, respectively. (E) Supernatant was harvested at the indicated times and viral titers were determined by TCID50 assay. Results were means from triplicate samples. Error bars represented standard deviations. ns = not significant.

To examine whether IL16 is critical for MHV68 lytic replication *in vitro*, WT and IL16 KO MEFs were isolated from E13.5 embryos and infected with MHV68-H2bYFP. Following infection with both a high multiplicity of infection (MOI) of 1 and a low MOI of 0.05, the expression of MHV68 vCyclin and lytic genes was detected by immunoblot analyses with MHV68 vCyclin antibody and MHV68 lytic serum, respectively. At an MOI of 1, both vCyclin and lytic antigen expression was detected at 12 hr and 24 hr post-infection and the expression levels were comparable between WT and IL16 KO MEFs; however, at an MOI of 0.05, MHV68 lytic gene expression detected by lytic serum was slightly reduced in IL16 KO MEFs compared to WT MEFs at 36 hr and 48 hr post-infection (Fig 3A). Next, we performed titration experiments to further analyze MHV68 replication over time at both high and low multiplicities in the WT and IL16 KO MEFs. In these viral growth assays, IL16 deficiency did not have a significant impact on MHV68 replication (Fig 3B), suggesting that IL16 is not essential for MHV68 efficient replication.

To assess the role of IL16 in lytic infection *in vivo*, WT and IL16 KO mice were infected with 50,000 PFU of MHV68-H2bYFP intranasally. Virus titers in the lungs of infected mice were quantified and the viral load was similar between MHV68-infected WT and IL16 KO mice at either day 4 or day 7 post-infection (Fig 3C). It’s worth noting that no viral load was detected in both WT and IL16 KO mice at day 9 post-infection, implicating that the infected mice might clear MHV68 from lung faster in our experimental setting than the observation by other groups [32, 33]. Altogether, our data suggest that IL16 does not affect MHV68 acute infection *in vivo*.

Considering the relatively low expression level of IL6 in MEFs, BHK21 cells with undetectable endogenous IL16 were overexpressed with IL16-expressing plasmid with Flag tag or vector alone for 24 hr, followed by MHV68 infection with a high MOI of 5 or a low MOI of 0.05. Immunoblot analyses did not show any difference in MHV68 lytic antigen expression regardless of IL16 expression (Fig 3D). Similarly, the viral growth curve assay revealed that there was no significant difference in MHV68 replication at both high and low MOI infection between vector and IL16-expressing cells (Fig 3E). These data further confirm that IL16 is not important for MHV68 lytic infection.

### IL16 inhibits MHV68 lytic reactivation in latently infected B cells independent of the secreted form of IL16

**Fig 4:**
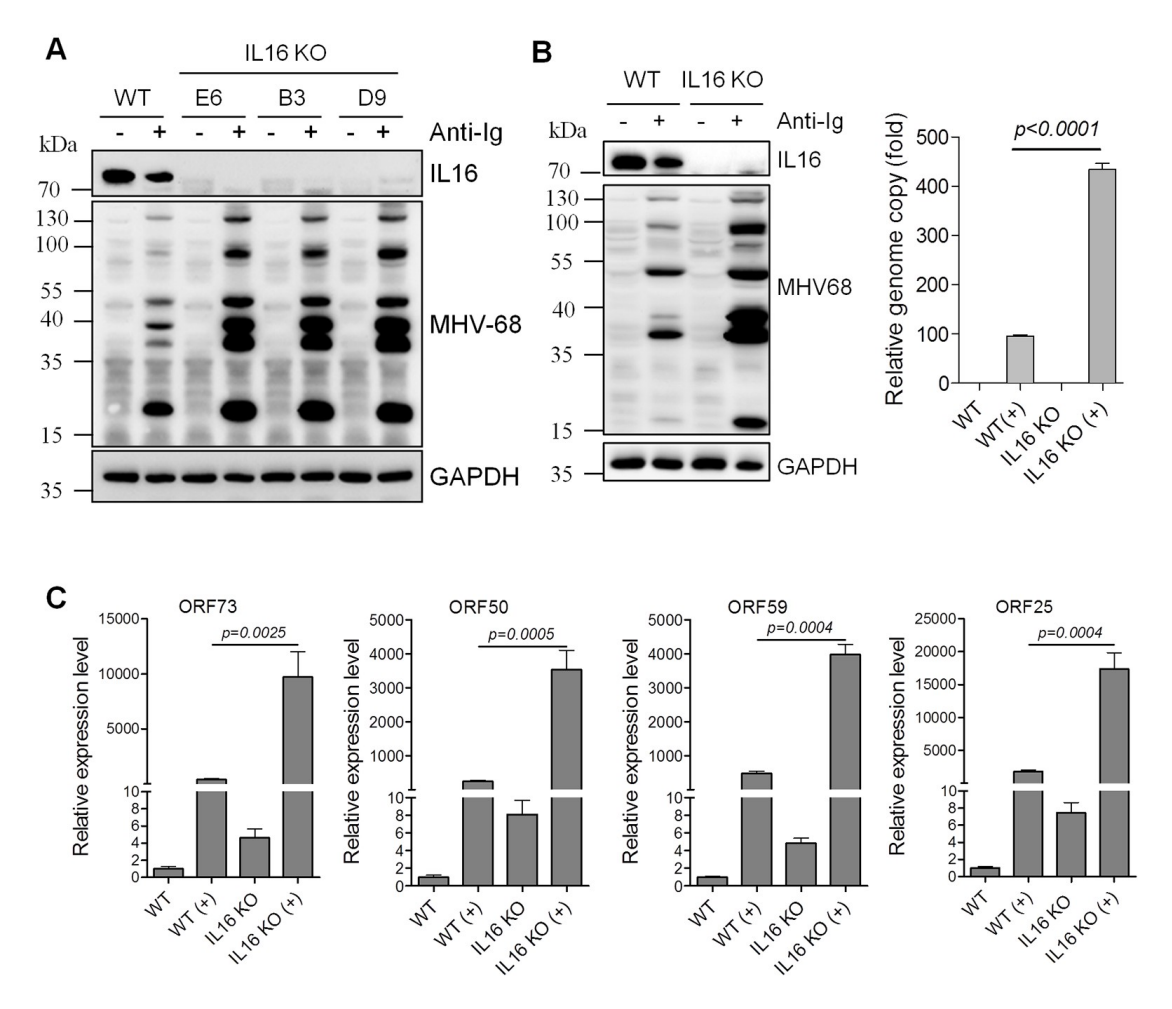
IL16 deficiency promotes MHV68 reactivation from B cell latency *in vitro*. (A) WT and three IL16 KO single clones were stimulated with (+) or without (-) anti-mouse Ig(G+M) (5 μg/mL) for 48 hr, respectively. Immunoblot analyses were performed with the indicated antibodies. GAPDH was used as a loading control. (B) WT and IL16 KO cells (clone E6) were stimulated with (+) or without (-) anti-mouse Ig(G+M) (5 μg/mL) for 48 hr. Immunoblot analyses were performed with the indicated antibodies. GAPDH was used as a loading control. MHV68 viral genome was determined by qPCR with the primers specific to the MHV68 ORF50 coding region. The relative copy of the MHV68 viral genome was normalized to GAPDH in each sample. (C) The mRNA expression of MHV68 viral gene ORF73, ORF50, ORF59, and ORF25 was determined by qRT-PCR. The relative RNA amount was normalized to GAPDH in each sample. Histograms represented the mean of three independent biological replicates ±SD, *p* value was determined by two-tailed unpaired t-test, *p*≤ 0.05 represents significance.

Taking into consideration of high-level expression of IL16 in MHV68 immortalized SL B cells in which viruses are tightly latent, we reasoned that IL16 potentially has an important function in MHV68 latency and reactivation. To test this, we used the CRISPR-Cas9 genome-editing system to generate IL16 KO SL-1 cells since IL16 expression was so abundant in SL-1 cells. Three single clones, E6, B3, and D9, were selected and confirmed by sequencing analyses. Surprisingly, all three clones showed the deletion of the IL16 genomic region between 95322 and 95564. Immunoblot analyses showed the efficient knockout of IL16 gene expression for all three single clones (Fig 4A). Surprisingly, IL16 KO greatly augmented MHV68 lytic gene expression detected by MHV68 lytic serum upon anti-Ig-induced reactivation in all single clones (Fig 4A). Clone E6 was used for all the following experiments. Next, we examined the viral genome and a series of viral gene expression. The MHV68 genome copy was significantly increased in IL16 KO SL-1 cells relative to WT cells (Fig 4B). Additionally, the expression of MHV68 representative latent gene ORF73, immediate early gene ORF50, early gene ORF59, and late gene ORF25 was also dramatically upregulated in IL16 KO cells as compared to that in WT cells (Fig 4C). Altogether, these data illustrate that IL16 inhibits MHV68 lytic reactivation from B cell latency.

**Fig 5:**
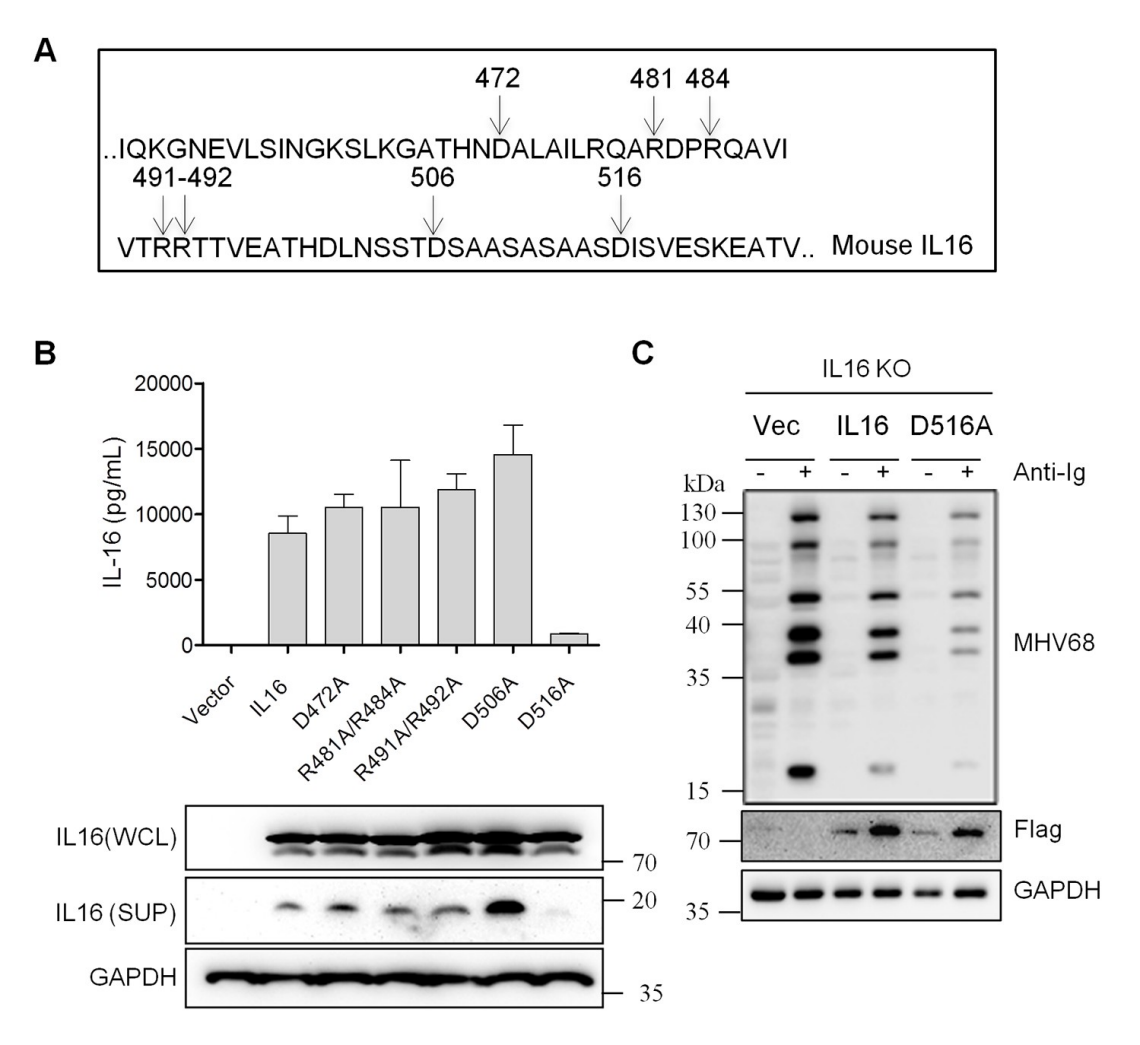
IL16 regulation of MHV68 reactivation is independent of the secreted form of IL16. (A) The diagram showed the potential cleavage sites of IL16. (B) 293T cells were transfected with IL16 or mutants with Flag tag. At 48 hr post-transfection, the supernatant was collected and subjected to IL16 ELISA assay; whole-cell lysates (WCL) and supernatant (SUP) were prepared and subjected to immunoblot analyses with the indicated antibodies. (C) IL16 KO SL-1 cells were transfected with vector (Vec), IL16-, or IL16(D516A)-expressing plasmid with Flag tag, followed by anti-mouse Ig(G+M) treatment for 48 hr. The whole-cell lysates were prepared and subjected to immunoblot analyses with the indicated antibodies.

Considering the capability of processing and secretion of IL16 [7], we next assessed whether the secreted form of IL16 accounted for the inhibition of MHV68 reactivation. Based on the previously reported human IL16 cleavage site and sequence alignment of human and mouse IL16 [7, 11], the potential cleavage sites of the C-terminal mouse IL16 were predicted, D472, R481R484, R491R492, D506, and D516 (Fig 5A). To abolish potential cleavage of IL16, the amino acids of targeted cleavage sites were changed to alanine (A) to yield point mutants, respectively. To examine whether these mutants would affect IL16 cleavage and secretion, 293T cells were transfected with the plasmids expressing IL16 and mutants with Flag epitope tag. After 48 hr post-transfection, the secreted form of IL16 was collected from the supernatant of transfected cell culture, subsequently detected by IL16 ELISA assay. Only the supernatant of IL16(D516A)-transfected cells showed a significant reduction of secreted IL16 level relative to IL16-transfected cells (Fig 5B). We next used IL16 antibody which can detect full-length IL16 and 20 kDa C-terminal secreted form of IL16 to examine IL16 expression in the whole-cell lysates and supernatant. Consistently, all mutants showed a similar level of full-length IL16 expression to WT IL16 in the whole-cell lysates, only IL16(D516A) supernatant showed undetectable C-terminal secreted IL16 in the supernatant, indicating that amino acid residue D516 could be critical for IL6 processing and secretion.

To determine how the mutant IL16(D516A) could affect MHV68 reactivation, IL16 KO SL-1 cells were transfected with the plasmids expressing WT IL16, mutant IL16(D516A), or vector alone, followed by reactivation induction with anti-mouse Ig(G+M). IL16 overexpression markedly reduced MHV68 lytic gene expression; surprisingly, IL16(D516A) overexpression led to a higher reduction in MHV68 lytic gene expression than WT IL16 overexpression (Fig 5C), suggesting that IL16 inhibition of MHV68 reactivation is independent of the secreted form of IL16, and the nonsecreted IL16 majorly contributes to its inhibitory effect.

### IL16 deficiency increases MHV68 reactivation from splenic latency *in vivo*

**Fig 6:**
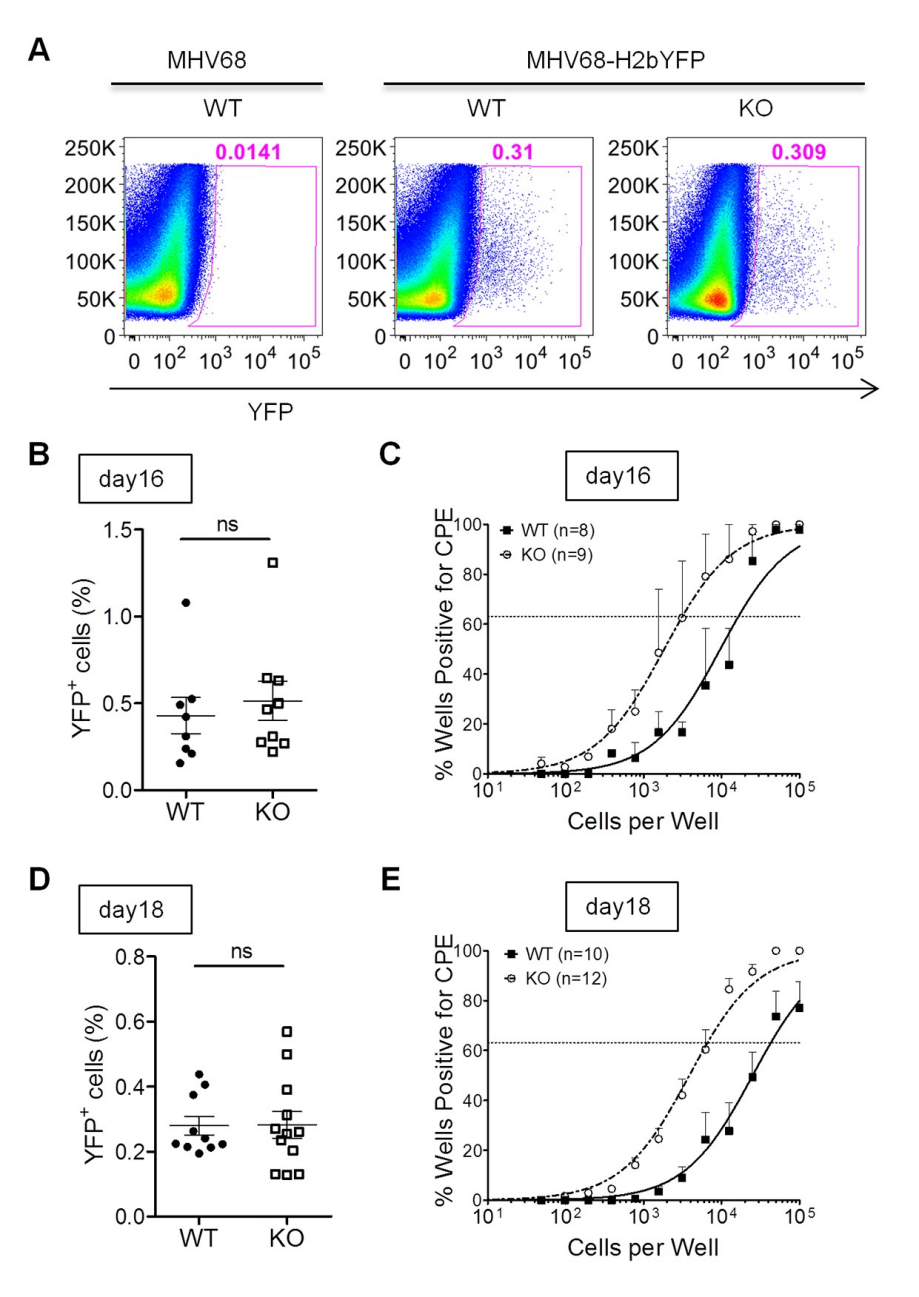
IL16 deficiency increases MHV68 reactivation from splenic latency. WT and IL16 KO mice were inoculated intranasally with 5×10^4^ PFU of MHV68-H2bYFP. Mice inoculated with 5×10^4^ PFU of WT MHV68 were used as a control to gate YFP+ cells. Splenocytes were isolated at day 16 and day 18 post-infection. (A) Representative flow plots showing the identification of MHV68-infected YFP+ cells. (B) Frequency of YFP+ cells at day 16 post-infection. Results were compiled from two independent experiments with 8–9 mice per group. Each symbol represented an individual mouse, and the horizon lines represented the mean frequency of infected cells. ns = not significant. (C) Frequency of splenocytes capable of reactivating virus by ex-vivo assay at day 16 post-infection. Serial dilutions of splenocytes were plated on MEFs and the presence of reactivating virus was determined by the presence of cytopathic effect (CPE). Representative results were from two independent experiments with 8–9 mice per group. (D) Frequency of YFP+ cells at day 18 post-infection. Results were compiled from two independent experiments with 10–12 mice per group. Each symbol represented an individual mouse, and the horizon lines represented the mean frequency of infected cells. ns = not significant. (E) Frequency of splenocytes capable of reactivating virus by ex-vivo assay at day 18 post-infection. Data were generated from two independent experiments, 5 to 6 mice per experiment per group.

The previous study has demonstrated that B cells are the major reservoir harboring latent MHV68 *in vivo* [34, 35], and play a crucial role in regulating reactivation of MHV68 from latency [28]. To analyze the role of IL16 in latency and reactivation *in vivo*, WT and IL16 KO mice were infected via intranasal inoculation with 50,000 PFU of MHV68-H2bYFP recombinant virus which has been shown to efficiently track virus-infected cells *in vivo* [31]. In parallel, WT mice were infected with WT MHV68 and used as staining controls to ensure that the observed YFP fluorescence was not due to autofluorescence. The splenocytes were harvested and MHV68-infected cells were monitored by flow cytometry. We observed a similar frequency of YFP-positive cells in the spleens of both WT and IL16 KO mice at day 16 post-infection (Fig 6A and 6B), suggesting that IL16 does not affect the establishment of MHV68 latency. Next, we performed an *ex vivo* reactivation assay and observed an about 5-fold increase in the frequency of the cells with reactivating viruses for the IL16 KO mice (1/3,232) compared to that for the WT animals (1/17,297) (Fig 6C), implicating that IL16 inhibits MHV68 reactivation from splenic latency. We further analyzed the splenocytes harvested at day 18 post-infection. Similarly, we did not observe any different frequency of YFP-positive cells in the spleens of both WT and IL16 KO mice at day 18 post-infection (Fig 6D), but similar to day 16 post-infection, we observed an about 6.5-fold increase in the frequency of the cells with reactivating viruses for the IL16 KO mice (1/6,607) compared to that for the WT animals (1/43,251) at day 18 post-infection (Fig 6E), further supporting that IL16 blocks MHV68 reactivation from splenic latency.

### IL16 deficiency increases MHV68-driven plasma cell frequency

**Fig 7:**
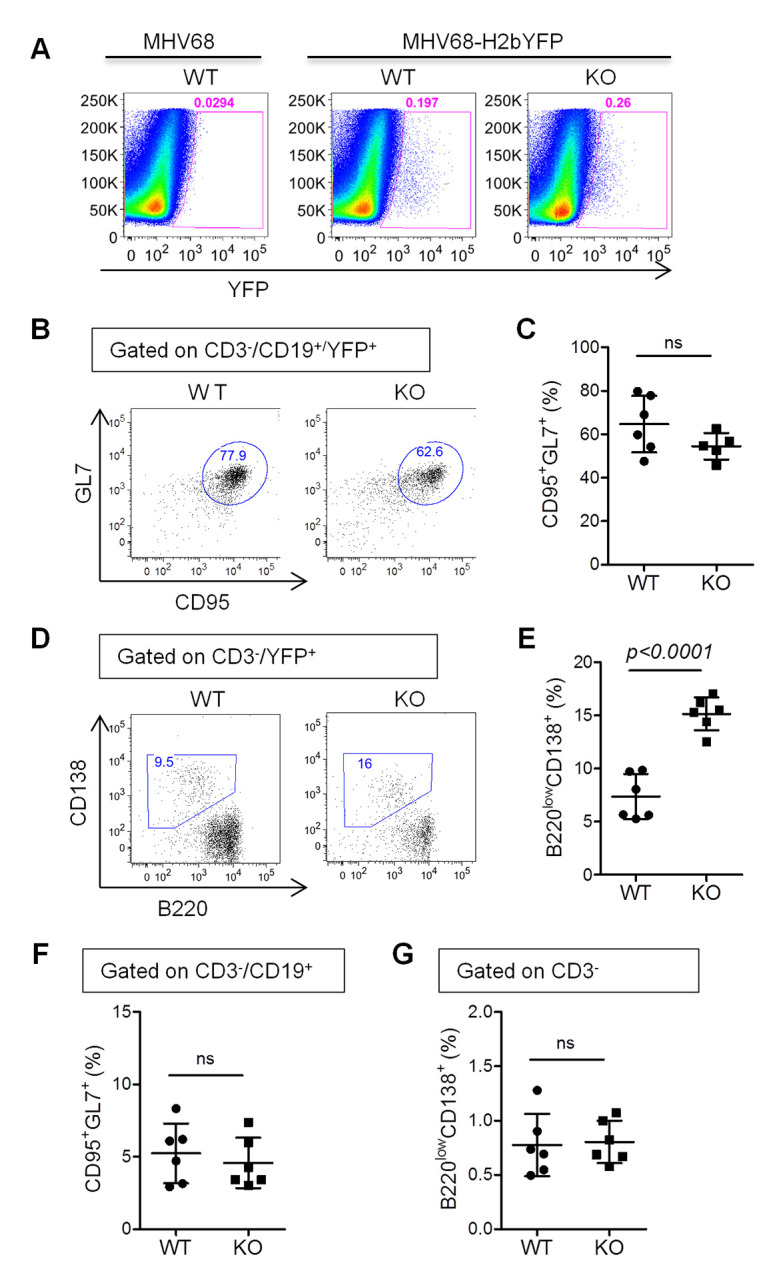
IL16 deficiency increases the frequency of MHV68-infected plasma cells. WT and IL16 KO mice were intranasally inoculated with 5×10^4^ PFU of MHV68-H2bYFP and splenocytes were harvested at day 16 post-infection. (A) Representative flow plots showing the identification of MHV68-infected YFP+ cells. (B) Representative flow plots of YFP+ germinal center B cells (CD19^+^CD95^+^GL-7^+^YFP^+^). (C) Quantitation of the percentage of YFP+ germinal center B cells. Each symbol represented an individual mouse, and the horizon lines represented the mean frequency. (D) Representative flow plots of YFP+ plasma cells (CD3^-^YFP^+^B220^low^CD138^+^). (E) Quantitation of the percentage of YFP+ plasma cells. Each symbol represented an individual mouse, and the horizon lines represented the mean frequency. (F) Quantitation of the percentage of total germinal center B cells (CD19^+^CD95^+^GL-7^+^). (G) Quantitation of the percentage of total plasma cells (CD3^-^B220^low^CD138^+^). ns = not significant. *p* value was determined by two-tailed unpaired t-test, *p*≤ 0.05 represents significance.

We have previously illustrated that plasma cells account for the majority of MHV68 reactivation from explanted splenocytes [36]. In light of the above-mentioned increase of MHV68 reactivation, we next assessed whether IL16 deficiency could similarly augment MHV68-driven plasma cell differentiation. To this end, we infected WT and IL16 KO mice with 50,000 PFU of MHV68-H2bYFP viruses intranasally. Virus-infected cells were analyzed by multicolor flow cytometry at day 16 post-infection (Fig 7A). The frequency of MHV68-infected germinal center B cells characterized by CD19^+^YFP^+^CD95^+^GL7^+^ was comparable between WT and IL16 KO mice (Fig 7B and 7C). However, the MHV68-infected plasma cell population characterized by CD3^-^YFP^+^B220^low^CD138^+^ was significantly increased in IL16 KO mice as compared to that in WT mice (Fig 7D and 7E). We also examined the total germinal center B cells and plasma cells in the infected animals, no significant difference was observed between WT and IL16 KO mice (Fig 7F and 7G). Taken together, these data suggest that IL16 deficiency promotes MHV68-driven plasma cell differentiation, consistent with elevating MHV68 reactivation from splenic latency observed above.

### IL16 deficiency increases CD4 T cell activation and IFN-γ-expressing Th1 cells

**Fig 8:**
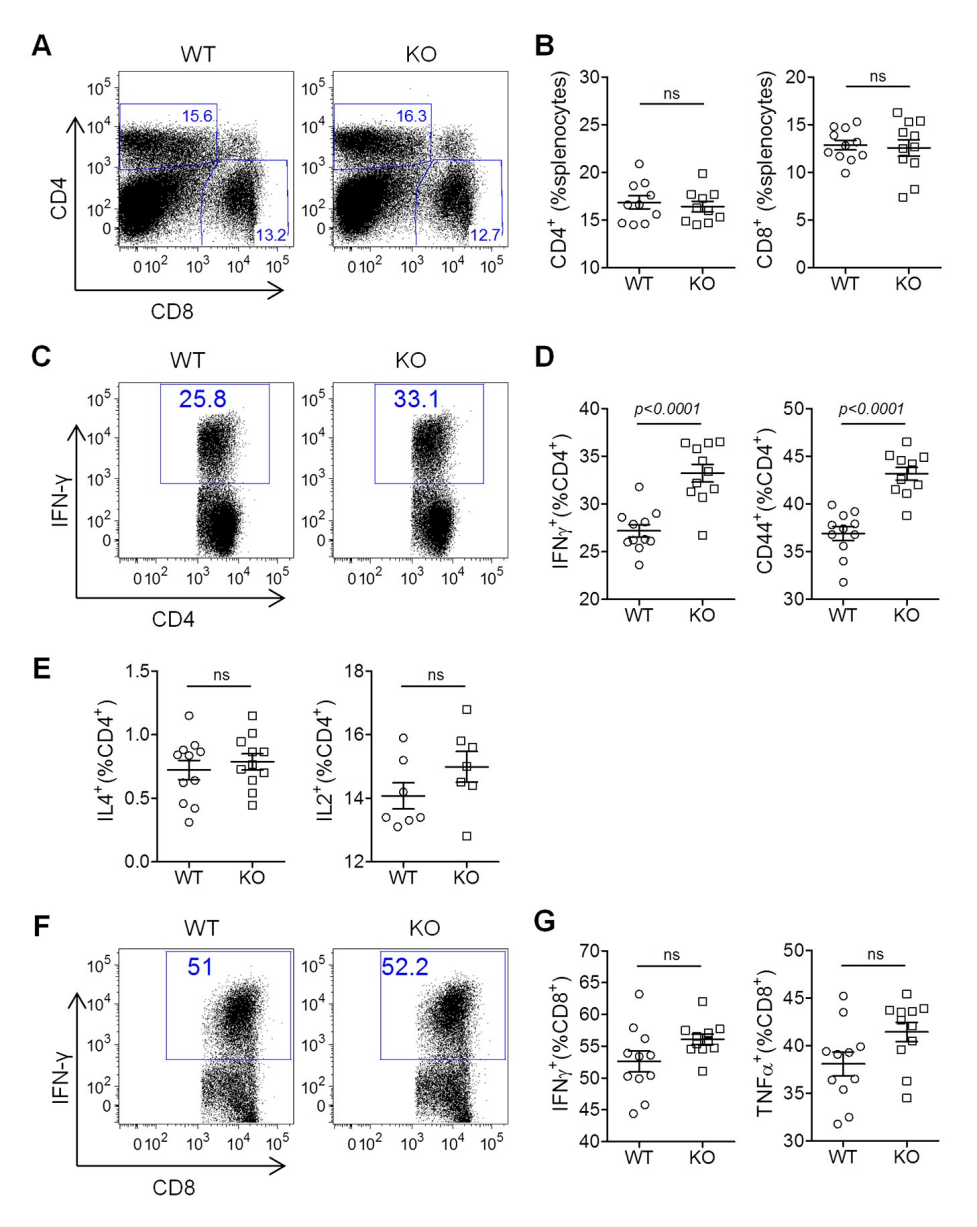
IL16 deficiency increases CD4 T cell activation and IFN-γ-expressing Th1 subset. WT and IL16 KO mice were inoculated intranasally with 5×10^4^ PFU of MHV68-H2bYFP. Splenocytes were isolated at day 16 post-infection and subjected to flow cytometry analyses. (A) Representative flow plots of CD4+ and CD8+ T cells from infected WT and IL16 KO mice. (B) Quantitation of the percentage of CD4+ and CD8+ T cells. Each symbol represented an individual mouse, and the horizon lines represented the mean frequency. (C) Representative flow plots of IFN-γ+CD4+ T cells from infected WT and IL16 KO mice. (D) Quantitation of the percentage of IFN-γ+CD4+ and CD44+CD4+ T cells. Each symbol represented an individual mouse, and the horizon lines represented the mean frequency. (E) Quantitation of the percentage of IL4+CD4+ and IL2+CD4+ T cells. Each symbol represented an individual mouse, and the horizon lines represented the mean frequency. (F) Representative flow plots of IFN-γ+ CD8+ T cells from infected WT and IL16 KO mice. (G) Quantitation of the percentage of IFN-γ+CD8+ and TNF-α+CD8+ T cells. Each symbol represented an individual mouse, and the horizon lines represented the mean frequency. ns = not significant. *p* value was determined by two-tailed unpaired t-test, *p*≤ 0.05 represents significance.

Previous studies have demonstrated that both CD4 T cells and CD8 T cells can control acute replication and latent infection of MHV68 [37, 38], whereas CD4 T cell control of acute and latent infection of MHV68 requires IFN-γ that has also been shown to regulate MHV68 reactivation [39]. In line with our recent data that IL16 deficiency enhances Th1 response and cytotoxic T cell response against influenza A virus infection [40], we next wanted to test whether IL16 deficiency affects T cell responses and consequently regulates MHV68 reactivation. WT and IL16 KO mice were infected with 50,000 PFU of MHV68-H2bYFP viruses intranasally, splenocytes isolated from infected mice at day 16 post-infection were subjected to flow cytometry analyses. Both CD4 and CD8 T cells showed similar frequency in WT and IL16 KO mice after MHV68 infection (Fig 8A and 8B), however, IFN-γ-expressing Th1 subset of CD4 T cells and CD44+ activated CD4 T cells were dramatically increased in IL16 KO mice as compared to WT mice (Fig 8C and 8D). We did not observe the difference for IL4-expressing Th2 subset and IL2-expressing CD4 T cells (Fig 8E). These data suggest that IL16 deficiency enhances CD4 T cell activation and Th1-biased response. However, we did not correspondingly observe the inhibition of MHV68 acute infection, latency, and reactivation in IL16 KO mice, suggesting that CD4 T cell response does not contribute to the above-demonstrated IL16 regulation of MHV68 reactivation. We next analyzed CD8 T cell responses and did not observe significant differences for IFN-γ-expressing CD8 T cells and TNF-α–expressing CD8 T cells between WT and IL16 KO mice at day 16 post-infection (Fig 8F and 8G). Altogether, these data demonstrate that IL16-mediated T cell responses are not involved in IL16 regulation of MHV68 reactivation.

### IL16 modestly inhibits MHV68 ORF50 proximal promoter activity

**Fig 9:**
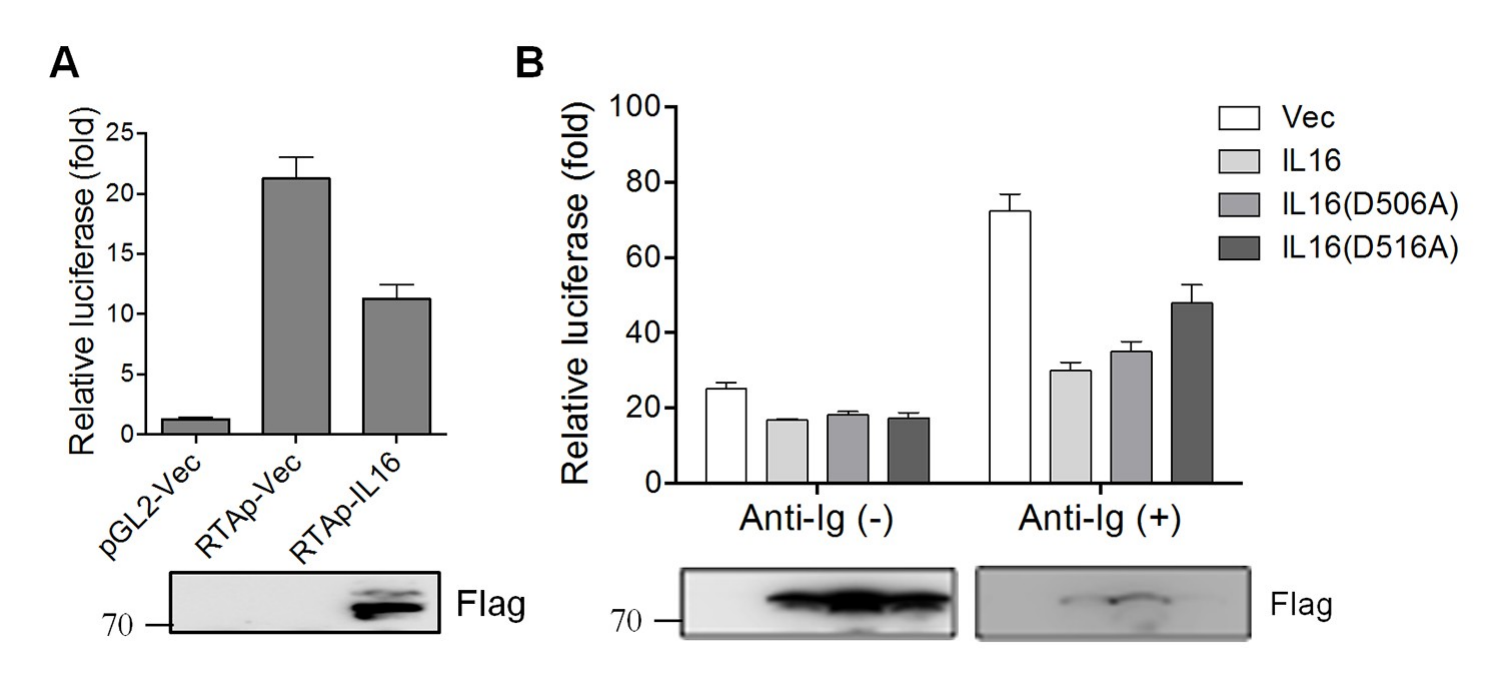
IL16 inhibits the RTA proximal promoter independent of the secreted form of IL16. (A) The murine M12 B lymphoma cells were transfected with renilla reporter, a luciferase reporter (pGL2) driven by RTA proximal promoter (RTAp), together with IL16-expressing plasmid with Flag tag or vector alone (Vec). Luciferase activity was normalized to renilla activity and Luciferase value was reported as fold increase in luciferase activity over basal promoter activity. Each sample was done in triplicate (two independent experiments). IL16 expression was detected by immunoblot with a Flag antibody. (B) M12 cells were transfected with RTAp together with vector (Vec), IL16-, IL16(D506A)- or IL16(D516A)-expressing plasmid with Flag tag. At 24 hr post-transfection, transfected cells were treated with (+) or without (-) anti-mouse Ig(G+M) (5 μg/mL) for 12 hr. IL16, IL16 (D506A), and IL16 (D516A) expression was detected by immunoblot with Flag antibody. Luciferase value was reported as fold increase in luciferase activity over basal promoter activity. Each sample was done in triplicate (two independent experiments).

MHV68 entry into the lytic replication cycle requires immediate-early gene ORF50, which encodes a transcriptional activator referred to as RTA. RTA’s transactivation function controls reactivation by turning on the expression of numerous replication-associated genes [41]. Given the inhibitory role of IL16 in MHV68 reactivation, we next examined whether IL16 could regulate RTA activation. Murine M12 B lymphoma cells were transfected with an expression construct expressing IL16 with a Flag epitope tag in conjunction with a luciferase reporter construct driven by RTA proximal promoter. IL16 expression led to about a 2-fold decrease in RTA transcriptional activity as compared to the basal promoter activity (Fig 9A), indicating that IL16 can inhibit RTA transactivation.

We next analyzed the effect of IL16 secreted form on MHV68 RTA activation, murine M12 B cells were transfected with RTA proximal promoter plasmid together with IL16, IL16(D506A), or IL16(D516A) in the presence or absence of anti-Ig stimulation. IL16 expression reduced the similar levels of RTA promoter activity as compared with IL16(D506) and IL16(D516A) expression in the absence of anti-Ig stimulation (Fig 9B). In the presence of anti-Ig, secretion-defective mutant IL16(D516A) which showed stronger inhibition of MHV68 reactivation exhibited a slightly less inhibitory effect on RTA promoter activity as compared to IL16 and IL16(D506A) (Fig 9B), suggesting that the secreted form of IL16 is dispensable for the inhibition of MHV68 RTA activation, and IL16 inhibition of RTA promoter activity might not be a crucial factor or the main determinant for the inhibitory role of IL16 in MHV68 reactivation.

### IL16 deficiency induces STAT3 Y705 de-phosphorylation and upregulates STAT3 S727 phosphorylation

**Fig 10:**
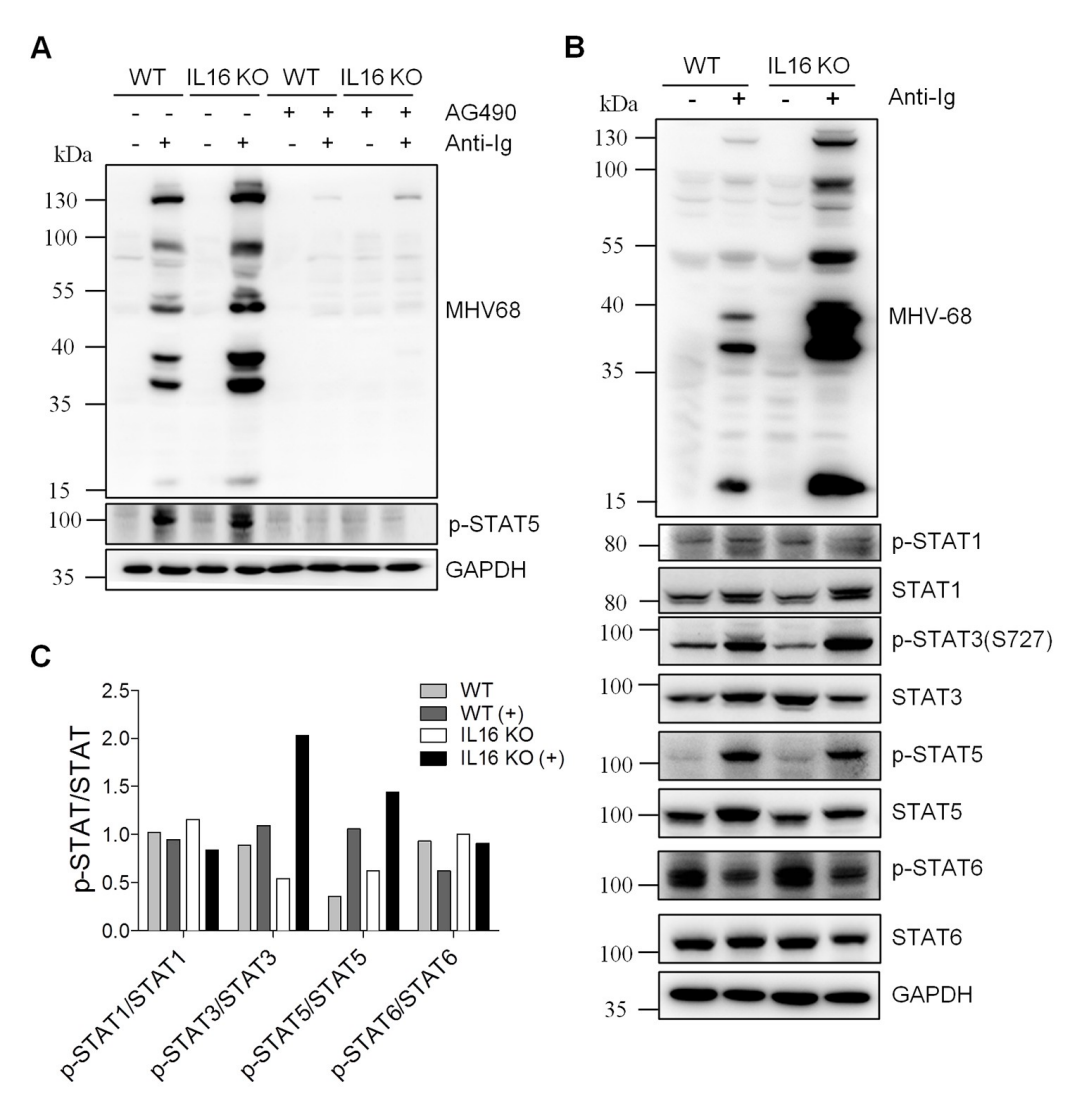
IL16 deficiency enhances STAT3(S727) phosphorylation. (A) WT and IL16 KO SL-1 cells were treated with (+) or without (-) 20 μM JAKs inhibitor AG490 for 1 hr, followed by stimulation with (+) or without (-) anti-mouse Ig(G+M) (5 μg/mL) for 48 hr. Immunoblot analyses were performed with specific antibodies as indicated. GAPDH was used as a loading control. (B) WT and IL16 KO SL-1 cells were treated with (+) or without (-) anti-mouse Ig(G+M) (5 μg/mL) for 48 hr. Immunoblot analyses were performed with specific antibodies as indicated. (C) Quantitation of phosphorylated STAT relative to total STAT based on immunoblot detection in C using the ImageJ image analysis software.

Given that cytokines binding to their receptors usually activate JAK-STAT signaling pathways and stimulates specific intracellular signaling to facilitate appropriate cellular responses [42]. Although the data presented above imply that the secreted form of IL16 did not play a role in MHV68 reactivation and the receptor of IL16 in B cells also remains unknown, we still could not rule out the possibility of JAK-STAT signaling participating in IL16 regulation of MHV68 reactivation from B cell latency. To resolve this, WT and IL16 KO SL-1 cells were treated with JAKs inhibitor AG490 in the presence or absence of anti-Ig. AG490 treatment blocked JAK-STAT signaling, evidenced by the inhibition of STAT5 phosphorylation (Fig 10A). Consequently, MHV68 lytic gene expression was completely suppressed in both WT and IL16 KO SL-1 cells (Fig 10A), suggesting that JAK-STAT signaling pathways are not only important for MHV68 lytic reactivation from B cell latency, might also critical for IL16 inhibition of MHV68 reactivation.

**Fig 11:**
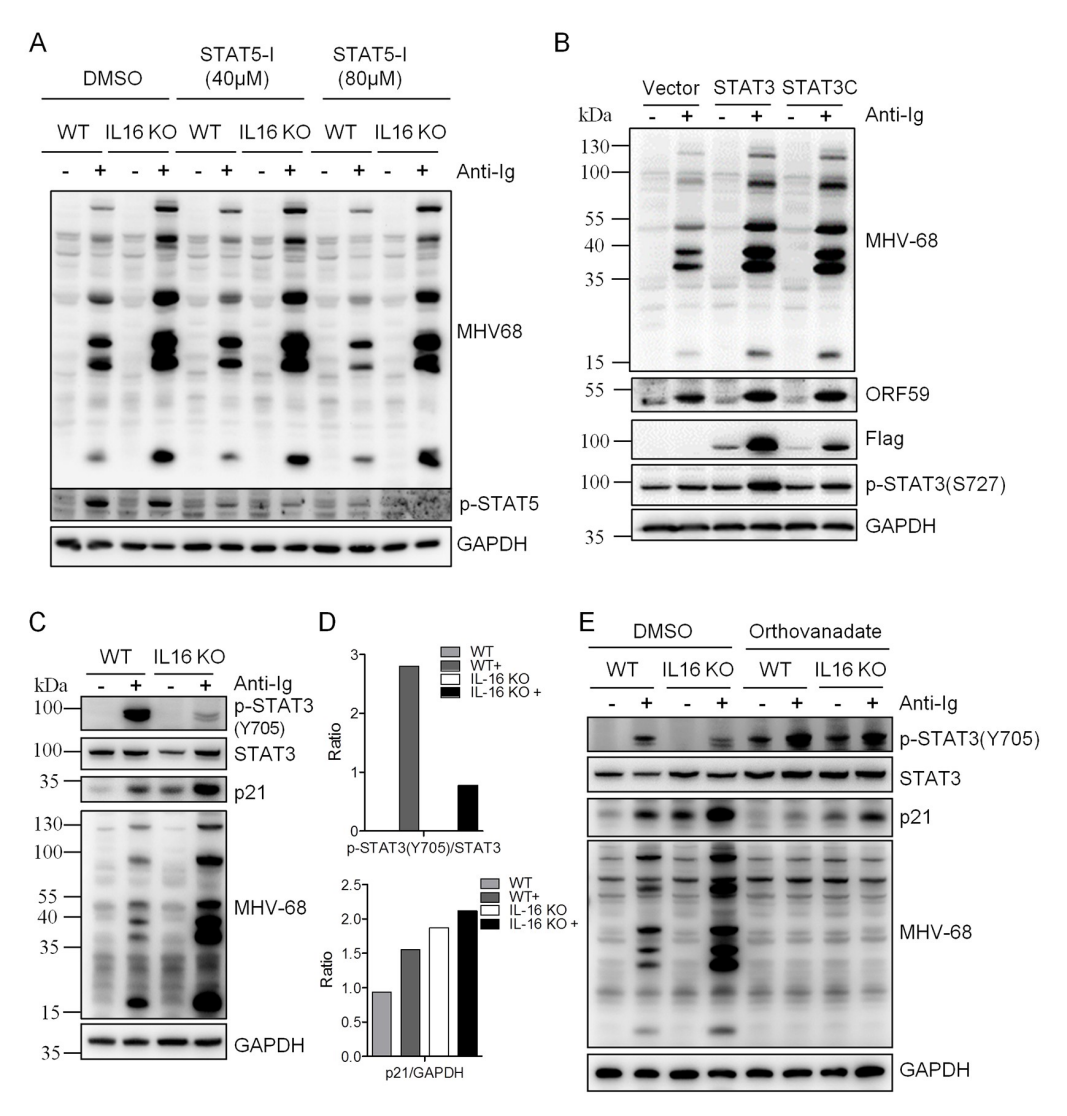
IL16-mediated STAT3(Y705) de-phosphorylation contributes to enhanced MHV68 reactivation. (A) WT and IL16 KO SL-1 cells were pretreated with DMSO, 40 μM, or 80 μM STAT5 inhibitor (STAT5-I) for 1 hr, followed by stimulation with anti-mouse Ig(G+M) (5 μg/mL) for 48 hr. Immunoblot analyses were performed with specific antibodies as indicated. (B) SL-1 cells were transfected with vector, STAT3-expressing plasmid, or STAT3C-expressing plasmid with Flag tag, followed by anti-mouse Ig(G+M) (5 μg/mL) treatment for 48 hr. Immunoblot analyses were performed with the indicated antibodies. (C) WT and IL16 KO SL-1 cells were treated with (+) or without (-) anti-mouse Ig(G+M) (5 μg/mL) for 48 hr. Immunoblot analyses were performed with the specific antibodies as indicated. (D) Quantitation of phosphorylated STAT3(Y705) relative to total STAT3 and p21 relative to GAPDH based on immunoblot detection in C using the ImageJ image analysis software. (E) WT and IL16 KO SL-1 cells were treated with DMSO or Orthovanadate (50 μM) in the presence or absence of anti-mouse Ig(G+M) (5 μg/mL) for 48 hr. Immunoblot analyses were performed with the specific antibodies as indicated.

To examine which STAT signaling was potentially involved in IL16 function, WT and IL16 KO SL-1 B cells were stimulated with anti-Ig to induce MHV68 reactivation and specific STAT activation was measured by the immunoblot analyses with the specific phosphorylation antibodies. Upon anti-Ig treatment, IL16 KO resulted in an about 2-fold increase in the level of phosphorylated STAT3(S727) over unphosphorylated STAT3 and a 1.4-fold increase in the level of phosphorylated STAT5 over unphosphorylated STAT5, whereas no change was observed for STAT1 and STAT6 phosphorylation (Fig 10B and 10C). We next used STAT5 specific inhibitor to treat WT and IL16 KO cells in the presence or absence of anti-Ig. We tested the inhibitor cytotoxicity and used the concentrations which showed no cytotoxicity. STAT5 phosphorylation was efficiently repressed when using 40 μM and 80 μM STAT5 inhibitors, however, MHV68 lytic gene expression was the same in control DMSO-treated and STAT5 inhibitor-treated WT or IL16 KO cells (Fig 11A), suggesting that STAT5 activation is not essential and important for MHV68 reactivation and IL16 function. Next, we assessed whether STAT3 activation observed in IL16 KO cells can enhance MHV68 reactivation. To test this, STAT3C, which is shown to function as a constitutively active form of STAT3 due to spontaneous dimerization [43], was generated and transfected into SL-1 cells, followed by anti-Ig stimulation. STAT3C overexpression dramatically augmented MHV68 lytic gene expression upon anti-Ig-induced reactivation, similar to the effect of STAT3 overexpression that showed the increased phosphorylation of STAT3(S727) (Fig 11B).

It has been reported that KSHV lytic cycle induced by TPA is mediated by an increase of STAT3(S727) phosphorylation and STAT3(Y705) de-phosphorylation which subsequently elevates p21 expression and KSHV lytic cycle induction [44]. Next, we wondered whether IL16 could regulate STAT3(Y705) de-phosphorylation. To test this, we analyzed STAT3(Y705) phosphorylation in WT and IL16 KO cells. Surprisingly, we observed a very similar phenotype in IL16 KO cells, STAT3(Y705) phosphorylation was significantly reduced, and p21 expression was also markedly upregulated (Fig 11C and 11D). Next, we used the pan-tyrosine phosphatase inhibitor orthovanadate to counteract STAT3 tyrosine de-phosphorylation, upregulation of p21 expression was inhibited by orthovanadate and MHV68 lytic gene expression was correspondingly blocked in IL16 KO cells (Fig 11E), suggesting that IL16 deficiency-mediated STAT3(Y705) de-phosphorylation and p21 upregulation contribute to enhanced MHV68 reactivation in IL16 KO cells, it is highly likely that IL16 regulates MHV68 reactivation through mediating STAT3(Y705)-p21 signaling cascade.

## Discussion

**Fig 12:**
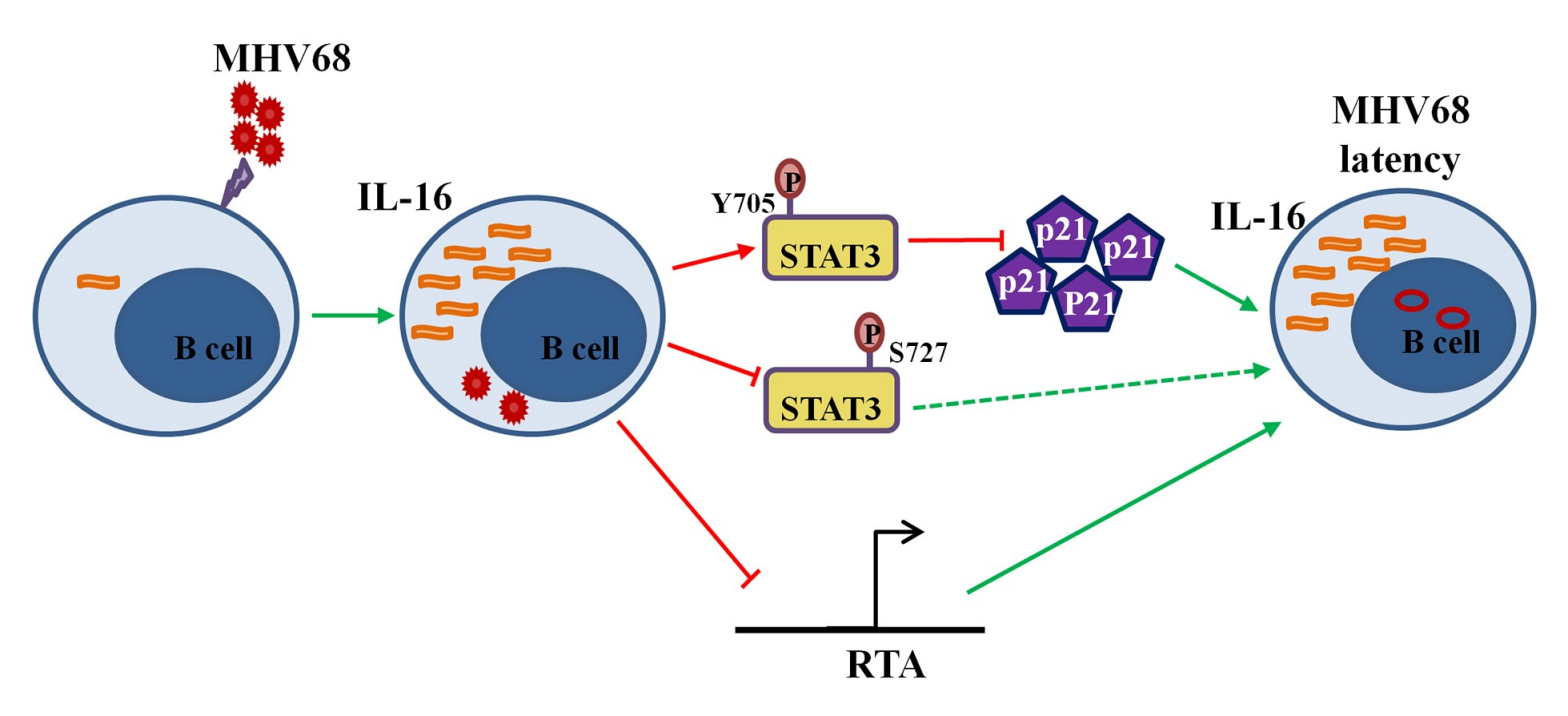
Working model of IL16 function during MHV68 infection. MHV68 infection induces IL16 production, which, in turn, increases STAT3(Y705) phosphorylation, subsequently reduces p21 expression, and inhibits MHV68 reactivation. *Meanwhile*, IL16 partially inhibits RTA promoter activity and STAT3(S727) phosphorylation, contributing to the inhibition of MHV68 reactivation. Ultimately, MHV68-induced IL16 helps to maintain MHV68 latency.

We have shown here that MHV68 infection induces expression of unique interleukin IL16, which has recently been shown to be a blood biomarker for “pan-viral” infection-induced systemic inflammation [45]. IL16 is first identified as a T cell chemoattractant factor and later shown to associate with various human diseases [46]. The previous studies have extensively focused on the function of IL16 in T lymphocytes and anti-HIV infection since IL16 shares the same CD4 receptor with HIV entry. The roles of IL16 in B cells and other virus infection remain largely unknown. Our current study demonstrates that MHV68-induced IL16 can increase STAT3(Y705) phosphorylation and reduce downstream p21 expression, at the meantime, inhibits STAT3(S727) phosphorylation and RTA activation, ultimately contributing to the inhibition of MHV68 reactivation from latently infected B cells (Fig 12). This funding shed light on the significant effects of IL16 on chronic viral infection.

Notably, most of the previous anti-HIV infection studies use the recombinant C-terminal peptide of IL16 instead of full-length IL16 [16–20], it is reasonable since the C-terminal peptide is secreted to self-aggregate and becomes a bioactive form of IL6 that can bind to CD4. However, our results show that the mutant IL16(D516A) with defective secreted IL16 exhibits considerably stronger inhibition of MHV68 reactivation than WT IL16 in SL-1 B cells, implicating that the secreted form of IL16 does not contribute to the inhibition of MHV68 reactivation. This raises an important question that different forms of IL16 exert very different functions, which is also evidenced by the previous studies of pro-IL16 function in T cell cycle and growth regulation [9, 47, 48] and secreted bioactive IL16 function in T cell migration [49–51]. Even though the secreted form of IL16 does not play a role in MHV68 reactivation from latently infected B cells *in vitro*, we do not rule out its potential function *in vivo* since a wide range of host cellular factors regulate MHV68 infection of mice, including those involved in innate and adaptive immune responses. Whether the secreted form of IL16 plays a role in innate and adaptive immune responses needs to be further investigated. Generating IL16 secretion-defective knock-in mice might be a rational setting to dissect the function of the secreted IL16 bioactive form in MHV68 infection.

Given that JAK-STAT signaling is the universal intracellular pathway for the secreted cytokines binding to their receptors. Our data showed that IL16 inhibits MHV68 reactivation from latently infected B cells independent of its secreted form seems involving JAK-STAT signaling. IL16 deficiency in enhancing MHV68 reactivation showed a very similar phenotype to TPA-induced KSHV lytic replication. Although IL16 deficiency can increase STAT3(S727) phosphorylation which might also contribute to enhanced MHV68 lytic reactivation, it is still not conclusive and needs to be further investigated. However, IL16 deficiency-mediated STAT3(Y705) de-phosphorylation and p21 expression upregulation appear to be the major contribution for IL16 regulation of MHV68 reactivation, as evidenced by the treatment of tyrosine phosphatase inhibitor orthovanadate. How IL16 mediates STAT3(Y705) de-phosphorylation and p21 expression upregulation remains to be resolved in the future study.

Constitutive activation of STAT3 and increased expression of STAT3 have been well demonstrated in EBV-related cancers and KSHV tumors. STAT3 was found to be constitutively active, typically phosphorylation at Y705, in EBV-positive nasopharyngeal cell carcinoma [52–54], EBV-positive B cell lymphomas [55–57], and KSHV tumors [58], supporting the role of STAT3 activation in gammaherpesvirus-related cancers. Furthermore, STAT3 is a key cellular regulator for latent and lytic phases of gammaherpesviruses [59–62]. In line with these reports, our observation that IL16 promotes STAT3(Y705) phosphorylation and inhibits MHV68 reactivation indicates that MHV68-induced IL16 might contribute to MHV68 immortalization and latency maintenance through mediating STAT3(Y705) phosphorylation.

The previous cytokine profile study following MHV68 infection shows that spleen from infected mice produces a high-level of IL6 and IFN-γ, whereas *in vitro* infection of naïve splenocytes induces IL16 and IL10 [63]. Our current study firstly shows that MHV68 infection also induces a unique cytokine IL16 that has not been detected before. Which viral gene of MHV68 is responsible for IL16 induction is under investigation. Concerning the capability of EBV EBNA2 to induce IL16 expression from microarray expression profiling analysis [30], it would be plausible for MHV68 to encode certain viral genes inducing IL16. The data presented here emphasize the importance of IL16 in the regulation of MHV68 reactivation from B cell latency. Although various human B cell lines are shown to constitutively express and secrete IL16 [3], very few works have been done to characterize the role of IL16 in B cells so far. Based on the involvement of IL16 in MHV68-driven plasma cell regulation, it’s rational to speculate that IL16 might be important for B cell responses. This provides us the impetus to illustrate IL16 function in B cells in the future study. Altogether, our results provide strong evidence that the virus takes advantage of the host by inducing host cell factors to inhibit viral reactivation, ultimately facilitating the maintenance of viral latency and contributing to viral pathogenesis.

## Materials and methods

### Ethics statement

All the infection protocols were approved by the Animal Care and Use Committee of Institut Pasteur of Shanghai (Protocol number: P2018044). All animal care and use protocols were performed in accordance with the Regulations for the Administration of Affairs Concerning Experimental Animals approved by the State Council of People’s Republic of China.

### Cell lines and reagents

MHV68-immortalized SL-1 and SL-3 cells were generated in our previous study [29] and cultured in RPMI 1640 (Gibco) supplemented with 5% heat-inactivated fetal bovine serum (FBS, Gibco) and 1% penicillin-streptomycin (Gibco) as previously described [29]. Murine M12 B lymphomas cell line was originally provided by Dr. David Schatz (Yale University School of Medicine) and cultured in RPMI 1640 containing 10% FBS and 1% penicillin-streptomycin. Mouse embryonic fibroblasts (MEFs) were isolated from day 13.5 pregnant C57BL/6 mice. BHK21 and NIH3T12 cells were from Dr. Samuel H. Speck (Emory University). MEFs, BHK21, and NIH3T12 cells were cultured in complete Dulbesco’s modified Eagle’s medium (DMEM, Gibco) containing 10% FBS and 1% penicillin/streptomycin.

STAT5 inhibitor 573108 and JAKs inhibitor InSolution AG490 (658411) were from EMD Millipore Corp. AffiniPure F(ab’) Fragment goat anti-mouse Ig(G+M) (115-006-068) was obtained from Jackson Immuno Research. Puromycin was from InvivoGen. Sodium orthovanadate (S2000) was from Selleck.

### Virus, Mice, and infection

MHV68-H2bYFP virus was from Dr. Samuel Speck (Emory University) and used for mice infection. Virus propagation and titer determination were performed in NIH3T12 cells as previously described [64].

IL16 KO mice were C57BL/6 background and generated by Shanghai Model Organisms Center, Inc with the CRISPR-Cas9 editing system. Each mouse was inoculated intranasally with 50,000 pfu of MHV68-H2bYFP virus that was diluted in 20 μL of PBS.

### Plasmids and transfection

The MHV68 RTA promoter (RTAp) plasmid containing the 410-bp proximal promoter region was from Dr. Samuel Speck (Emory University) and described previously [65]. The IL16-expressing plasmid with a C-terminal Flag epitope tag was generated by inserting PCR-amplified mouse IL16 coding fragment into the restriction enzyme *Eco*RI and *Bam*HI sites of pLVX-puro vector (Clontech). The following forward and reward primers were used: 5’-GGAATTCATGGACTATAGCTTTGATATCACTGCTGAAG-3’ and 5’-CGGGATCCTTACTTGTCATCGTCGTCCTTGTAGTCTGAGTCTGCAGAAGCTGTTGTCTG-3’. IL16 mutants were generated by overlapping PCR with the specific primers. The primers for D472A were: 5’-CTACCCACAATGCTGCCCTAGCTATCCTTC-3’ and 5’-GAAGGATAGCTAGGGCAGCATTGTGGGTAG-3’; the primers for R481A/R484A were: 5’-CGCCAAGCTGCGGACCCAGCGCAAGC-3’ and 5’-GCTTGCGCTGGGTCCGCAGCTTGGCG-3’; the primers for R491A/R492A were: 5’-GATTGTCACCGCGGCGACAACTGTGGAG-3’ and 5’-CTCCACAGTTGTCGCCGCGGTGACAATC-3’; the primers for D506A were: 5’-AACTCCTCTACTGCTTCCGCAGCCTC-3’ and 5’-GAGGCTGCGGAAGCAGTAGAGGAGTT-3’; the primers for D516A were: D516: 5’-CTTCAGCAGCCAGTGCCATTTCTGTAGAATC-3’ and 5-GATTCTACAGAAATGGCACTGGCTGCTGAAG-3’.

IL16 KO plasmids lentiCRISPRv2-IL16-1 and lentiCRISPRv2-IL16-2 were generated with lentiviral CRISPR/Cas9 system. Two pairs of sgRNA targeting IL16 were cloned into a lentiCRISPRv2 vector (Addgene) following the protocols provided by Addgene. Two pairs of sgRNA sequences targeting mouse IL16 gene were: CACCGCCCAGTTAGGTGTCATCCCT and AAACAGGGATGACACCTAACTGGGC; CACCGATGCACATCTGTAGGGACAA and AAAC TTGTCCCTACAGATGTGCATC.

### Cytokine array and enzyme-linked immunosorbent assay (ELISA)

1×10^6^ SL-1 and SL-3 cells were cultured for 24 hr, whole-cell lysates were prepared and culture supernatant was collected for cytokine array analyses with Mouse Cytokine Array, Panel A (ARY006, R&D Systems) according to the manufacturer’s instruction.

ELISA for IL16 serum level was performed following the manufacturer’s instruction with Mouse IL16 ELISA kit (DuoSet ELISA development system, DY1727, R&D Systems). Briefly, serum was collected from the infected mice at day 16 post-infection and subsequently subjected to ELISA based on the manufacturer’s protocol. Absorbance at 450 nm was measured using a microplate reader (VARIOSKAN FLASH, Thermo Fisher).

### Flow cytometry

Cell surface staining was carried out by standard procedures and analyzed on Beckon Dickinson LSR Fortessa (BD Biosciences). Data were analyzed using Flowjo (Treestar Inc). The intracellular staining was performed according to the manufacturer’s instruction (Cytofix/Cytoperm Plus Fixation/permeabilization kit, 554715, BD Biosciences).

The antibodies used were: CD3-BV510(145-2c11), CD4-BV711(GK1.5), CD8-BV650(53–6.7), CD19-PE, CD19-percp-cy5.5(1D3), CD3e-Pacific BlueTM (500A2), CD95-PE-cy7 (Jo2), CD138-APC(281–2), IgD-APC(11-26C.2a), IgG2a.κ Isotype-PE, IL-16-PE and CD21/CD35-FITC(7G6) were purchased from BD Biosciences. B220-PE, B220-PE-cy7, B220-FITC (RA3-6B2) and GL-7-eFlour(GL-7) were purchased from Invitrogen. CD19-APC(eBio1D3), IgM-eFlour 450(ll141), CD23-PE-cy7(B3B4), CD43-PE(R2/60) and CD117(c-kit)-APC(2B8) were purchased from eBioscience. CD4-APC(GK1.5), TNF-ɑ-PE-cy7(MP6-XT22) were purchased from BD Biosciences. CD8-PE-cy7(53–6.7), IFN-γ-APC(XMG1.2), CD44-PE(IM7), IL-4-APC(11B11), IL-2-PE(JES6-5H4) were purchased from eBioscience.

### Generation of IL16 KO cell lines

The CRISPR-Cas9 genome-editing system was used to generate IL16 KO SL-1 cells. 2×10^6^ SL-1 cells were transfected with 4 μg lentiCRISPRv2-vector, lentiCRISPRv2-IL16-1, or lentiCRISPRv2-IL16-2, respectively, using the Amaxa Nucleofector II system (Lonza) according to the manufacturer’s instruction. After 48 hr post-nucleofection, the nucleofected cells were selected with puromycin at a concentration of 2 μg/mL for two weeks and subsequently cloned by limiting dilution culture. Individual clones were subjected to sequencing analyses to confirm the deletion of the IL16 target region and immunoblot analyses to detect IL16 KO efficiency.

### qRT-PCR, qPCR, and Western blot

qRT-PCR and qPCR were used to measure MHV68 viral gene expression and genome copy, respectively, as previously described [65]. Total RNA was isolated from the treated cells using TRIzol reagent (Invitrogen) as described in the manufacturer’s instructions. qRT-PCR was carried out with the specific primers corresponding to MHV68 ORF73, ORF50, ORF59, and ORF25 [65]. To measure MHV68 genome copy, genomic DNA was isolated with a Tianamp Genomic DNA kit (DP304-02; TianGen) and used for qPCR with the primers corresponding to ORF50. IL16 primers used in qRT-PCR were: 5’-CTGCATGGTGACAAGCCTCT-3’ and 5’-CCAGGCTTCAAACCGGGTAA-3’.

Western blot was performed following the standard procedure. The antibodies used were as follows: p21(SX118) (sc-53870), GAPDH (sc-32233) and Actin (sc-1616) were from Santa Cruz Biotechnology. Mouse IL16 Antibody (842373) was from R&D Systems. Anti-Flag-HRP (A8592) was from Sigma. Stat1 (9172), p-Stat1 (Y701) (7649P), Stat3 (9132), p-Stat3 (S727) (9134P), p-Stat3 (Tyr705) (9145), Stat5 (9363), p-Stat5 (Y694) (4322P), Stat6 (9362), p-Stat6 (Y641) (936P) were from Cell Signaling Technology. Polyclonal MHV68 antiserum to detect lytic antigen was obtained from C57BL/6 mice 6 weeks post-infection. vCyclin antibody was described previously [66].

### Luciferase assay

Luciferase assays were carried out as previously described [67]. MHV68 RTA promoter and Renilla reporter plasmids were transfected into M12 cells by nucleofection (Lonza) for 24 hr and subsequently stimulated with or without 5 μg/mL anti-mouse Ig(G+M) for 12 hr. Luciferase assays were carried out in triplicate. Luciferase activity was measured using the Dual-Luciferase Reporter Gene Assay kit (Beyotime Biotechnology) according to the manufacturer’s instructions and normalized to Renilla activity.

### Reactivation assay

The frequency of MHV68 reactivation from latency was detected by limiting dilution *ex vivo* assay analysis. Briefly, splenocytes were harvested from infected mice at day 16 post-infection, and single-cell suspensions were generated. After erythrocytes were lysed with ammonium chloride, the cells were resuspended in complete DMEM and plated onto MEF monolayers in 96-well tissue culture plates in a series of twofold dilutions, starting at 10^5^ cells per well with splenocytes. After 21 days, wells were scored microscopically for the presence of the cytopathic effect.

### Statistical analyses

Statistical analysis was performed in Prism (Graph Pad Software). The data were reported as means and standard deviations (SD). Differences between groups of research subjects were analyzed for statistical significance with two-tailed Student t-tests. A *P* value of 0.05 was considered significant.
